# More Than What You Eat: A Review on the Association Between Childhood Maltreatment and Elevated Adult BMI

**DOI:** 10.1007/s13668-024-00558-4

**Published:** 2024-06-26

**Authors:** Carmelle Wallace, Richard Krugman

**Affiliations:** 1 Kempe Center for the Prevention and Treatment of Child Abuse and Neglect, Department of Pediatrics, University of Colorado School of Medicine, Aurora, CO USA; 2 Section of Emergency Medicine, Department of Pediatrics, University of Colorado School of Medicine, Aurora, CO USA

**Keywords:** Child maltreatment, Obesity, Child abuse, Elevated BMI, ACEs

## Abstract

**Purpose of Review:**

Obesity is an overwhelmingly common medical entity seen in the adult population. A growing body of research demonstrates that there is a significant relationship between child maltreatment and adult obesity.

**Recent Findings:**

Emerging research demonstrates a potential dose–response relationship between various types of child abuse and adulthood BMI. Recent work also explores the potential role of the hypothalamic–pituitary–adrenal (HPA) axis, and other hormonal mediators such as sex-hormone binding globulin and leptin. There are also studies that suggest factors such as depression and socioeconomic and environmental influences mediate this relationship. Comorbidities that have been reported include cardiovascular and metabolic disease, diabetes, and insulin resistance. Preliminary work also demonstrates potential gender and racial disparities in the effect of abuse on adulthood obesity.

**Summary:**

In this narrative review, we summarize the existing work describing the different child maltreatment types (physical, sexual, emotional, verbal, and child neglect) and their relation to adult obesity, what is known about a potential dose-response relationship, potential mediators and pathophysiology, comorbidities, and preliminary work on gender and racial/ethnic disparities. We review the limited data on interventions that have been studied, and close with a discussion of implications and suggestions for clinicians who treat adult obesity, as well as potential future research directions.

## Introduction

Obesity is an increasingly prevalent public health problem with significant medical, mental health sequelae and societal implications including cardiovascular disease, diabetes, cancer, premature death, and an estimated economic health care burden of $172.74 billion annually [1]. The initial landmark study on Adverse Childhood Experiences (ACEs) introduced the concept of the relationship between childhood trauma and obesity, exploring ACEs and their association with high-risk behaviors and diseases with life-limiting consequences including as obesity, heart disease, cancer [2]. Accordingly, current medical evidence suggests that obesity as a medical condition is best understood as a multifactorial condition, with dynamics between genetics, health behaviors, environmental conditions, and exposures such as childhood maltreatment, trauma, and ACEs [3]. A breadth of medical literature has further explored the implications of toxic childhood stress and the specific association between childhood maltreatment and elevated body mass index (BMI) and obesity. This narrative review summarizes specifically what is known about the types of child maltreatment that have been implicated, timing and the dose–response relationship, neurobiology, mediators, comorbid outcomes, and emerging research on disparities and interventions.

## Child Maltreatment Types

Child maltreatment, inclusive of physical and sexual abuse, psychological and verbal abuse, and child neglect have been described in association with obesity. This has been noted by several large cross-sectional surveys of adults with obesity [4–8]. Relative rates of maltreatment type vary by cohort. A meta-analysis conducted by Hemmingson and colleagues confirmed that multiple types of abuse (including physical, psychological, sexual) are all significantly associated with adult obesity, with no difference found between retrospective and prospective study designs [9].

While literature elucidating potential differences in effects by maltreatment type is limited, multiple studies suggest nonuniformity, and the conclusions vary widely by sample and study type. Several studies suggest child sexual abuse (CSA) is associated with adulthood obesity while not finding the same association with physical abuse. In a large representative U.S. cross-sectional sample, Fuemmeler and colleagues found an association between CSA and obesity in men, but not in women [10]. Similarly, another large study found CSA and child emotional abuse associated with increased BMI in women but did not find the same association for other abuse types and among men [11].

Conversely, a prospective cohort study where children with substantiated physical abuse, CSA, and neglect were followed into adulthood 30 years later, physical abuse was found to be a significant predictor of increased adult BMI scores while sexual abuse and neglect were not [12]. A systematic review evaluating interpersonal violence in childhood as a risk factor for obesity also found consistent associations in the majority of studies between physical abuse and obesity, though sexual abuse was also noted, and neglect and emotional abuse was not explored [13].

Some studies have also suggested that childhood emotional abuse or neglect rather than physical or sexual abuse are significantly associated with adult obesity. One longitudinal study found emotional abuse and neglect to be risk factors for obesity [14]. Mason et al. also found this in their cohort, noting emotional abuse to have the strongest association with BMI with no association noted for sexual or physical abuse [15].

## Timing and Dose–Response Relationship

Current research explores the patterns of obesity development over the lifespan of children who experience maltreatment. Several studies suggest that obesity emerges early in adulthood. A prospective cohort found that women with histories of child abuse had higher BMI’s in early adulthood, and a large cross-sectional study found overall significantly lower age of onset of adulthood obesity in victims of childhood abuse [4, 11, 16, 17]. Furthermore, higher rates of BMI increase over time have also been described [16, 18].

A few studies have also found a dose–response relationship between childhood abuse and obesity. Two studies have found that victims of the co-occurrence of physical and sexual abuse demonstrate a stronger association with obesity risk, while another longitudinal cohort found that more severe abuse correlated with a higher obesity risk [15, 16, 19].

## Neurobiology

Several neurobiological mechanisms have been studied as potential mediators. The hypothalamic–pituitary–adrenal (HPA) axis is the most studied. The HPA axis is the primary neuroendocrine mediator for stress-responses and has been studied in multiple abused, neglected, and trauma-exposed populations [3]. The role of cortisol has been evaluated in several studies specifically looking at child maltreatment and obesity. One such study tested hair cortisol and cortisone levels and found that higher levels of abuse was associated with higher cortisone levels, suggestive of chronic stress levels. However, a mediating effect was not found for cortisol or cortisone between childhood maltreatment and BMI [20]. In two additional studies, cortisol response was tested via salivary testing and was found to be blunted in girls and women who had been sexually abused [21, 22]. Furthermore, poly-victimization was linked to lower cortisol response and higher BMI [21].

Other potential biological mediators include sex-hormone binding globulin (SHBG), which has previously been implicated in mediating central adiposity in women [23]. One study demonstrated lower SHBG levels in women with a history of childhood trauma [13]. Leptin and inflammatory mediators have also been suggested, with a study by Danese and colleagues measuring leptin, BMI and c-reactive protein in maltreated children. Leptin provides negative feedback to downregulate adiposity by decreasing energy intake and increasing energy expenditure. They found that maltreated children had lower leptin levels with increasing inflammation and BMI [24].

## Mental Health and Environmental Mediators

Childhood maltreatment has many sequelae, some of which are mediators of the effect on adult obesity. Mental health outcomes are often inter-related. Multiple studies have demonstrated depression as a mediating factor, especially in females [25–28]. Anxiety has also been described, specifically in women in one study [29, 30]. Post-traumatic stress disorder (PTSD) has also been widely described as a result of childhood maltreatment, though notably has not consistently been demonstrated as a mediator [31, 32].

Certain eating disorders also have an expected causal link with obesity, and studies indicate that it may also function as a mediator of the child maltreatment effect. Some studies link physical abuse and disordered eating including coping-motivated eating as mediators of obesity [10, 17]. On the other hand, emotional neglect and abuse have also been demonstrated to increase eating disorders, including night eating and binge eating syndromes [33, 34]. Substantiated child maltreatment in general has also been found to be associated with high dietary fat intake, food addiction, and binge eating [35, 36]. Preliminary work has also queried the impact of child maltreatment on impulsivity and has been found to moderate sexual abuse and obesity [37].

Socioeconomic and environmental factors are well established risk factors for obesity. Whether they function as mediators of the effect between child maltreatment and obesity is less clear, though a large survey study found that 15–20% of the association between ACEs and adult health risks (including obesity) could be attributable to socioeconomic factors [38]. Another large cross-sectional study found that childhood maltreatment was associated with adulthood obesity (as measured by weight-to-height ratio) but the socioeconomic factors mitigated the association in women but not in men [39].

## Comorbid Outcomes

One of the measured comorbid outcomes of childhood ACEs in general includes cardiovascular and metabolic disease [2, 40]. Similarly, victims of childhood maltreatment suffer not only from increased obesity, but also cardiometabolic comorbidities. Abuse has been associated with adulthood diabetes and insulin resistance [4, 41]. Hyperlipidemia and lower HDL levels have also been reported [4, 14, 42].

## Disparities

Data exploring gender disparities are limited and is even more sparse with regards to racial disparities. Nonetheless, preliminary work suggests potential differences, though trends are not well established. Several studies suggest that the association between CSA and BMI is present in females but not males [18, 43, 44]. Neglect and physical abuse have been described to demonstrate a stronger effect on BMI in females compared to males, and emotional abuse has shown a similar pattern [25, 43]. One study has reported the opposite effect, finding that CSA in males was associated with increased obesity compared with females [10]. In contrast, other studies have also found no gender difference [9, 45].

With regards to race, very few studies have evaluated potential disparities. One analysis of a large, longitudinal survey study found no race or ethnicity effect on the association between childhood physical abuse and obesity [45]. Another cross-sectional study conversely did find a difference, demonstrating a statistical relationship between child maltreatment and obesity in Whites but not in Blacks [46]. In a study with large proportions of Asian, Native Hawaiian and Pacific Islander representation, no race/ethnicity effect was found [47]. There are additional studies where samples consist primarily of one or a few particular races or ethnicities, but do not offer comparisons.

## Interventions

Research on secondary prevention interventions targeting the effect of child maltreatment on obesity is sparse. Tavernier and colleagues studied a behavioral weight-loss intervention and found that physical abuse predicted a percent lower weight loss compared to emotional abuse or no abuse history [48]. Another study explored the role of mentorship in promoting resilience in child maltreatment survivors and found that mentorship was associated with decreased depression symptoms, but also conversely increased obesity [49]. While tertiary and primary interventions for obesity have been widely studied, the potential role of child maltreatment-targeted interventions remains poorly understood.

## Conclusions

The body of research demonstrating a relationship between child maltreatment and obesity in adulthood is relatively recent. However, it is continuing to grow and holds significance for the myriad of types of clinicians who care for adults with obesity. While the exact physiology and ecological influences for this relationship are not yet fully understood, the data is robust enough that we suggest that clinicians caring for adult patients with overweight and obesity *specifically* query about histories of child abuse and neglect in a trauma-sensitive manner. For example, asking if the patient has ever been to therapy or struggled with mental health or substance use may open the door to further conversation about why. Clinicians may also consider asking a direct, but sensitively worded question about whether there are any life stressors that relate to their childhood, or even consider asking the patient themselves if there was any childhood trauma such as abuse that they perceive to relate to their current struggle with obesity. Clinicians should also consider when referrals or even clinic integration with mental/behavioral health colleagues may provide the holistic care needed to address the multifactorial issue of adult obesity.

Future work should explore in-depth the physiology and socio-ecological mechanisms for these trends, as this understanding may hold implications not only for current management but also potential primary prevention efforts that could be directed towards children who experience child maltreatment. There is also a paucity of literature on whether universal screening versus individualized, tailored history-taking would be the most effective in assisting clinicians in uncovering these histories and understanding its implications for the current management of their patient’s obesity. We also suggest careful research exploring the cultural and social implications of having these conversations as clinicians, and how clinicians may most sensitively and non-judgmentally approach their patients about this sensitive topic. Finally, data supporting the effectiveness of various interventions, as discussed above, is preliminary. Therefore, future directions should also work towards identifying which approaches for addressing childhood maltreatment may yield long-term positive outcomes.
